# In situ forming microporous gelatin methacryloyl hydrogel scaffolds from thermostable microgels for tissue engineering

**DOI:** 10.1002/btm2.10180

**Published:** 2020-09-02

**Authors:** Nicole Zoratto, Donatella Di Lisa, Joseph de Rutte, Md Nurus Sakib, Angelo Roncalli Alves e Silva, Ali Tamayol, Dino Di Carlo, Ali Khademhosseini, Amir Sheikhi

**Affiliations:** ^1^ Department of Bioengineering University of California, Los Angeles Los Angeles California USA; ^2^ Center for Minimally Invasive Therapeutics (C‐MIT), University of California, Los Angeles Los Angeles California USA; ^3^ California NanoSystems Institute (CNSI), University of California, Los Angeles Los Angeles California USA; ^4^ Department of Drug Chemistry and Technologies Sapienza University of Roma Rome Italy; ^5^ Department of Informatics, Bioengineering, Robotics and System Engineering University of Genova Genoa Italy; ^6^ Department of Chemical Engineering The Pennsylvania State University Pennsylvania USA; ^7^ Experimental Biology Center (NUBEX), University of Fortaleza (UNIFOR) Fortaleza Ceará Brazil; ^8^ University of Connecticut Health Center Farmington Connecticut USA; ^9^ Jonsson Comprehensive Cancer Center, University of California, Los Angeles Los Angeles California USA; ^10^ Department of Radiological Sciences David Geffen School of Medicine, University of California, Los Angeles Los Angeles California USA; ^11^ Department of Chemical and Biomolecular Engineering University of California, Los Angeles Los Angeles California USA; ^12^ Terasaki Institute for Biomedical Innovation Los Angeles California USA; ^13^ Department of Biomedical Engineering The Pennsylvania State University University Park Pennsylvania USA

**Keywords:** GelMA microgels, in situ forming microporous hydrogels, MAP gels, microfluidics, microporous hydrogels, thermostable GelMA microbeads, tissue engineering

## Abstract

Converting biopolymers to extracellular matrix (ECM)‐mimetic hydrogel‐based scaffolds has provided invaluable opportunities to design in vitro models of tissues/diseases and develop regenerative therapies for damaged tissues. Among biopolymers, gelatin and its crosslinkable derivatives, such as gelatin methacryloyl (GelMA), have gained significant importance for biomedical applications due to their ECM‐mimetic properties. Recently, we have developed the first class of in situ forming GelMA microporous hydrogels based on the chemical annealing of physically crosslinked GelMA microscale beads (microgels), which addressed several key shortcomings of bulk (nanoporous) GelMA scaffolds, including lack of interconnected micron‐sized pores to support on‐demand three‐dimensional‐cell seeding and cell–cell interactions. Here, we address one of the limitations of in situ forming microporous GelMA hydrogels, that is, the thermal instability (melting) of their physically crosslinked building blocks at physiological temperature, resulting in compromised microporosity. To overcome this challenge, we developed a two‐step fabrication strategy in which thermostable GelMA microbeads were produced via semi‐photocrosslinking, followed by photo‐annealing to form stable microporous scaffolds. We show that the semi‐photocrosslinking step (exposure time up to 90 s at an intensity of ~100 mW/cm^2^ and a wavelength of ~365 nm) increases the thermostability of GelMA microgels while decreasing their scaffold forming (annealing) capability. Hinging on the tradeoff between microgel and scaffold stabilities, we identify the optimal crosslinking condition (exposure time ~60 s) that enables the formation of stable annealed microgel scaffolds. This work is a step forward in engineering in situ forming microporous hydrogels made up from thermostable GelMA microgels for in vitro and in vivo applications at physiological temperature well above the gelatin melting point.

## 1 INTRODUCTION

Hydrogels sourced from natural and/or synthetic materials have leveraged technologies pertinent to water treatment, energy storage, food security, and healthcare.^1^, ^2^, ^3^, ^4^, ^5^, ^6^, ^7^ Granular hydrogel scaffolds have introduced emerging opportunities for developing in vitro tissue/disease models as well as in vivo therapies in regenerative medicine^8^, ^9^, ^10^, ^11^ owing to their unique structural features, particularly interconnected pores that promote cellular infiltration and enhance tissue remodeling.^12^, ^13^ The success of such scaffolds relies on their capability to overcome some of the key shortcomings of bulk hydrogels, mainly unconnected nanoscale pores that are 2–3 orders of magnitude smaller than the cell size. In fact, the assembly of microgel building blocks in granular hydrogels enables the fabrication of scaffolds in situ (e.g., during surgery) that have interconnected microscale pores originated from the void spaces between the linked microspherical building blocks.^12^, ^14^ Specifically, the formation of interstitial spaces between the building blocks in a jammed state yields a three‐dimensional (3D) porous network with high pore connectivity, facilitating cell migration and diffusive/convective transport of nutrients and oxygen.^10^, ^15^ Moreover, the scaffold building blocks can be fabricated with well‐controlled size and may have a soft, reversibly deformable (elastic) nature that allow them to be injected through needles or catheters and, hence, enabling minimally invasive procedures.^9^, ^12^, ^16^

Usually, the fabrication of in situ forming microgel‐based scaffolds is a two‐step process involving the aqueous stabilization of individual submillimeter‐sized hydrogel beads. Several strategies have been developed to crosslink individual beads and stabilize them prior to annealing, which include physical and/or chemical gel formation via phase transition, chemical crosslinking, enzymatic linkage, and/or photocrosslinking.^8^, ^14^, ^17^, ^18^, ^19^ Furthermore, several biomaterials have been used to develop granular scaffolds, including gelatin,^8^, ^20^ hyaluronic acid,^14^ and polyethylene glycol^12^, ^21^ among which methacryloyl‐modified gelatin (gelatin methacryloyl [GelMA]) has gained attention due to a facile synthesis procedure and tunable physicochemical and biological properties, such as arginylglycylaspartic acid peptide motifs that facilitate cell adhesion, tissue adhesiveness, and physical gel formation below physiological temperature.^22^, ^23^, ^24^

Droplet microfluidics technology is a promising approach for fabricating hydrogel microbeads with well‐controlled sizes (low polydispersity) and shapes (sphericity ~1).^8^, ^13^, ^20^, ^25^, ^26^ We have recently developed a high‐throughput, two‐step method for the stabilization and annealing of GelMA microbeads to fabricate microporous 3D hydrogel scaffolds in situ.^8^, ^20^, ^27^ In this strategy, we use the temperature‐induced physical crosslinking of GelMA microbeads, produced as a water‐in‐oil emulsion using droplet microfluidics, followed by the chemical crosslinking and annealing of beads via free radical photo‐annealing. The physical crosslinking of GelMA droplets is conducted by decreasing the temperature to ~4°C, enabling the beads to hold their shape while being transferred to an aqueous medium wherein the annealing process takes place. Hence, the working temperature should be maintained at 4°C during the process of particle transfer from the oil to the aqueous phase and later during the in situ annealing process. Using physical crosslinking, we were able to demonstrate robust annealing while avoiding the complexities of other approaches, for example, use of high power UV sources to overcome quenching of free radicals in oxygen‐rich oils^28^ or the addition of organic bases^13^ to initiate crosslinking. Despite these advantages, issues associated with the phase transition (melting) of gelatin at physiological temperature^29^ may limit the biomedical applications of physically crosslinked GelMA beads, mainly during handling in vitro (e.g., cell culture) or injection/annealing in vivo. As an example, fabricating cell‐laden GelMA microporous scaffolds requires microgel–cell mixture to undergo annealing at low temperatures to preserve beads; however, most cells demand physiological temperatures near the body temperature. At a low temperature, cells are prone to a lowered rate of physiological processes and reduced membrane fluidity, leading to a change in intracellular pH, compromised biomacromolecular integrity, and cellular stresses.^15^

In this paper, we aim at engineering thermostable, annealable GelMA microbeads without further chemical modification of GelMA and the annealing process. We investigate how partial photocrosslinking of GelMA microgels in an oil phase affects the microgel stability at physiological temperature and their annealing capability. Our design criteria are based on tailoring the preannealing condition, particularly photocrosslinking time, to obtain semi‐photocrosslinked GelMA microbeads that hold their shape in aqueous media at 37°C for up to 24 hr and are able to undergo photo‐annealing to form stable GelMA microporous scaffolds with pore size and structure similar to scaffolds made up of freshly prepared, nonchemically crosslinked beads. We study the physical and biological properties of these scaffolds and compare them with the scaffolds assembled from physically crosslinked beads. This work introduces new routes to engineer in situ forming microporous GelMA scaffolds from the chemical assembly of thermostable GelMA microgel building blocks, which may have applications in tissue engineering and regeneration.

## 2 RESULTS

**FIGURE 1:**
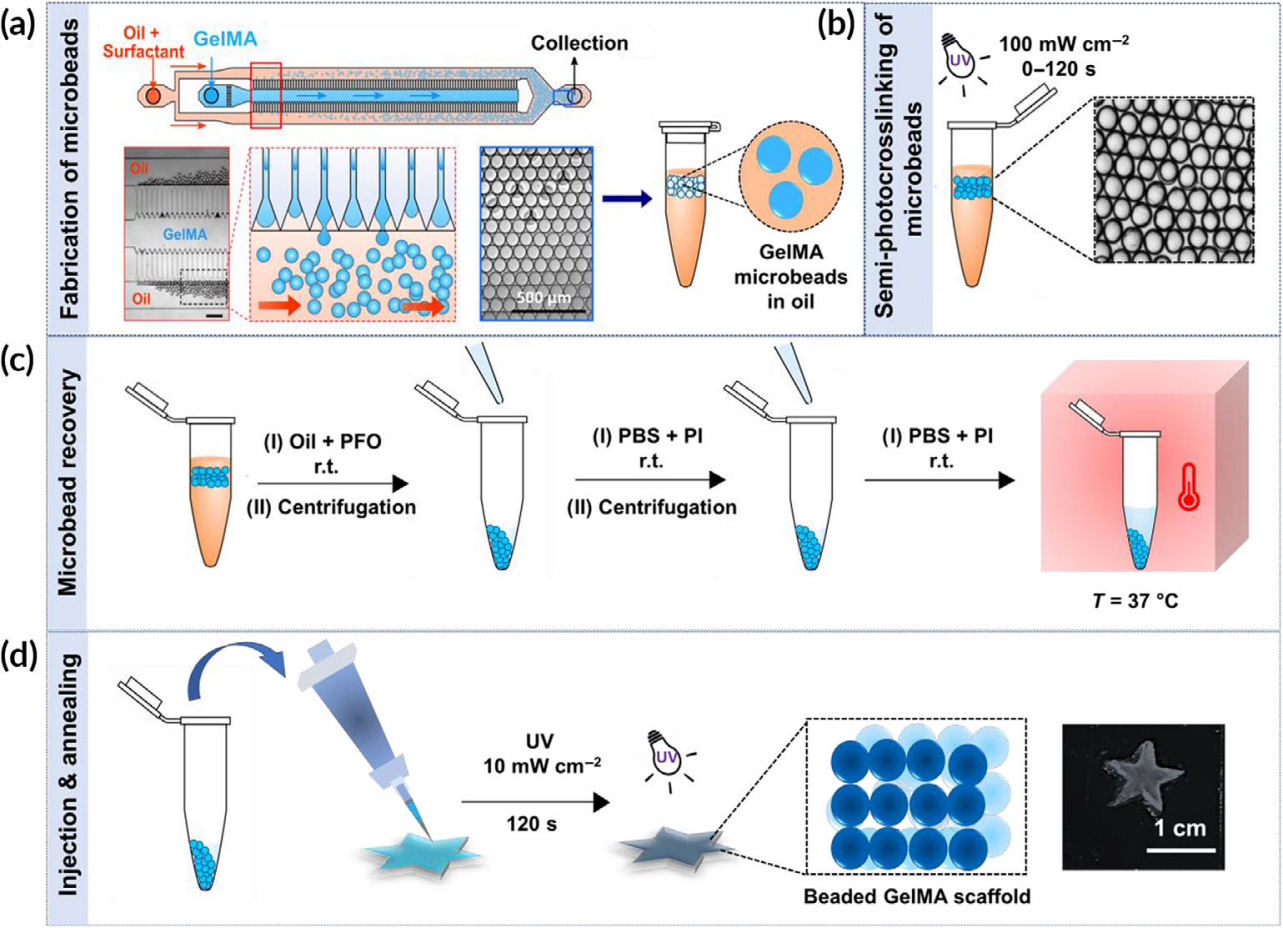
Workflow schematic for the fabrication of in situ forming gelatin methacryloyl (GelMA) microporous scaffolds from semi‐photocrosslinked GelMA microgels. (a) High‐throughput fabrication of GelMA microbeads using a step emulsification microfluidic device. (b) Stabilizing GelMA microgels in an oil phase via ultraviolet (UV)‐light‐mediated semi‐photocrosslinking at an intensity of ∼100 mW/cm^2^ for varying times: 0 s (control), 60, 90, and 120 s. (c) Microgel purification from the oil phase, followed by transfer to an aqueous phase and incubation in Dulbecco's phosphate‐buffered saline (DPBS) at 37°C for 30 min to assess the shape fidelity as a measure of thermostability. (d) Annealing the semi‐photocrosslinked GelMA microbeads through secondary UV light exposure at an intensity of ∼10 mW/cm^2^ for 2 min to form microporous hydrogel scaffolds

GelMA microbeads (diameter ~90 μm) were fabricated using a high throughput droplet microfluidic device based on our previous protocols.^8^, ^13^, ^20^ An aqueous GelMA solution (20% wt/vol) was injected into the droplet generator along with a continuous phase composed of oil and surfactant (0.5% PicoSurf™ in Novec 7500™) to generate water in oil droplets. The aqueous phase was composed of a mixture of GelMA and a photoinitiator (Irgacure 2959, 0.5% wt/vol) (Figure 1a). The microbeads were collected in the oil phase and partially crosslinked by ultraviolet (UV)‐light exposure at an intensity of ~100 mW/cm^2^ and wavelength of ~365 nm for varying periods **(0–120 s)** to induce varying degrees of crosslinking (Figure 1b). Lower UV light intensities resulted in a decrease in the production yield of thermostable microgels. The semi‐photocrosslinked microgels were then purified from the oil‐surfactant mixture using a biocompatible destabilizing agent (1H,1H,2H,2H‐perfluoro‐1‐octanol, PFO), as shown in Figure 1c, and were maintained at 37°C for 30 min to dissolve the noncrosslinked beads. Finally, the microgels were washed with Dulbecco's phosphate‐buffered saline (DPBS), packed via centrifugation, pipetted into a mold, and exposed to the UV light to initiate the annealing process and form microporous 3D scaffolds (Figure 1d). The photo‐annealing process was conducted using a much lower UV light intensity (~10 mW/cm^2^) to remain within a safe range of exposure intensity and time for mammalian cells.^30^, ^31^, ^32^

**FIGURE 2:**
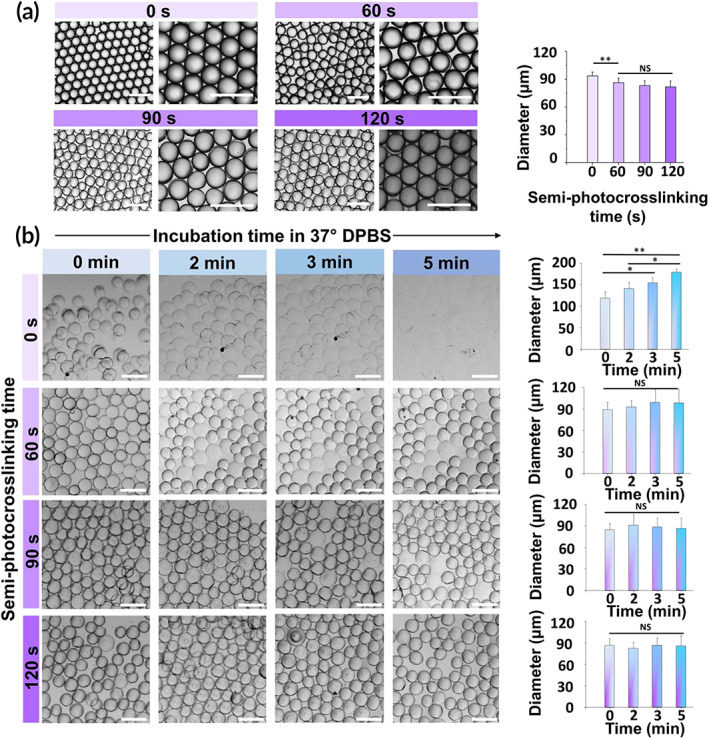
Effect of in‐oil semi‐photocrosslinking on the size, short‐term stability at physiological temperature, and swelling of gelatin methacryloyl (GelMA) (20% wt/vol) microgels. (a) Optical images and corresponding size of GelMA microgels produced in oil using a droplet microfluidic device as a function of in‐oil photocrosslinking time. (b) Thermal stability of semi‐photocrosslinked GelMA microgels over time as a function of photocrosslinking time. The scale bars represent 200 μm. For each condition, at least 4 snapshots containing 30 microbeads each were analyzes (120 microbeads in total for each condition)

UV‐light intensity and exposure time affect the GelMA photopolymerization process. Thus, we investigated the effect of exposure time on the crosslinking of GelMA microbeads at a high, constant exposure intensity (~100 mW/cm^2^) by varying the UV‐curing time from 0 to 120 s. Using a high UV intensity for bead stabilization in oil is acceptable as cells are introduced at a later step. Figure 2a shows the effect of photocrosslinking time on the shape of GelMA droplets in the oil phase. While the unexposed GelMA microgels attained a near‐perfect spherical shape, the exposure to UV light resulted in the formation of gel particles with slightly irregular surfaces. In addition, the chemical crosslinking of droplets in the oil phase significantly affected the GelMA bead size. While the step emulsification microfluidic device generated droplets with a diameter of ~93 ± 1 μm, after 60 s of UV light exposure, the bead size decreased to ~84 ± 1 μm, as shown in Figure 2a, and further increase in the crosslinking time did not significantly affect the microgel diameter. We speculate that the bead diameter is fixed by an equilibrium reached between radical generation and quenching by oxygen^28^ in the oil.

**FIGURE 3:**
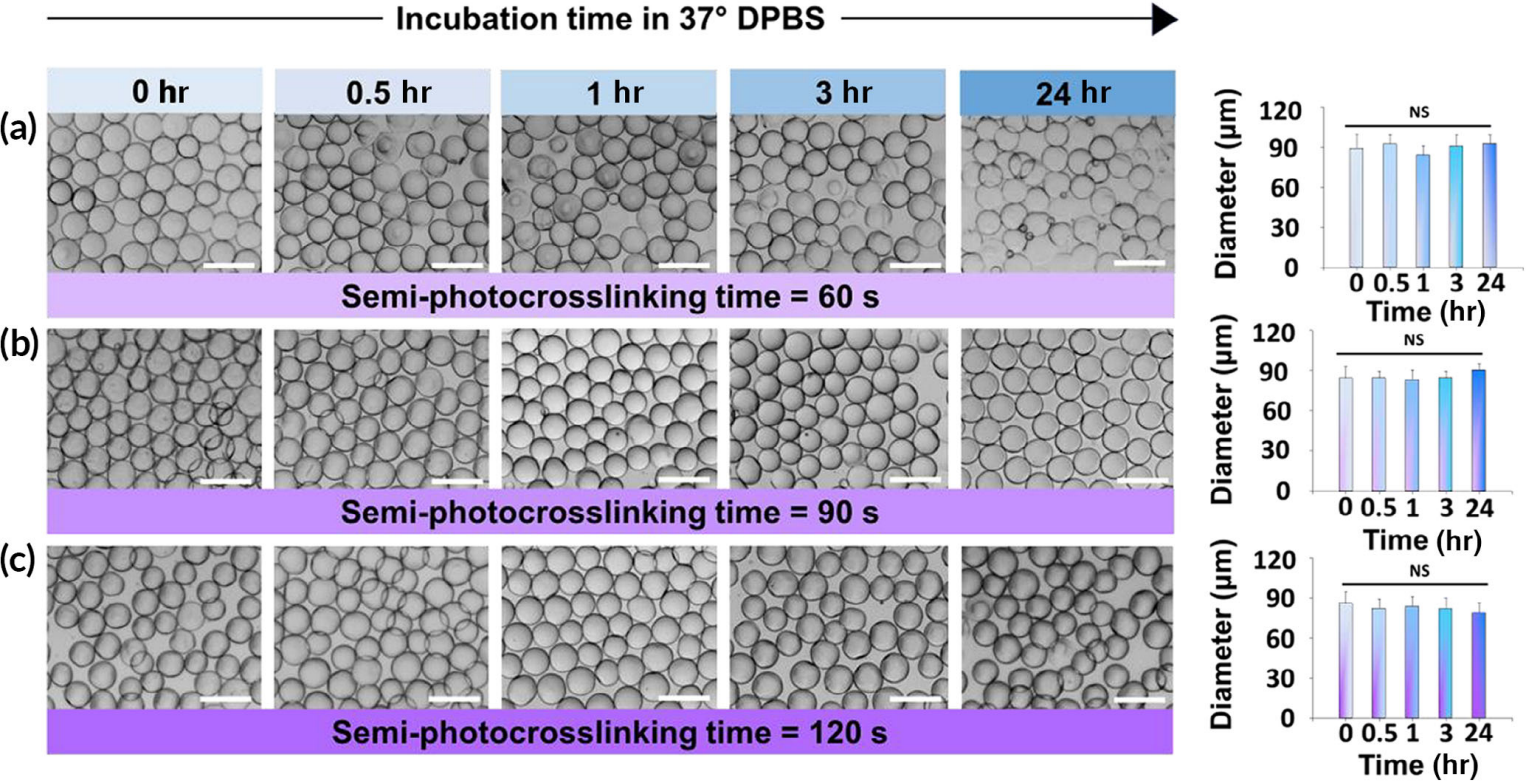
Long‐term thermostability and swelling of semi‐photocrosslinked gelatin methacryloyl (GelMA) microgels. Microbeads were photocrosslinked in oil for (a) 60 s, (b) 90 s, and (c) 120 s, followed by transferring to Dulbecco's phosphate‐buffered saline (DPBS) and incubating at 37°C for up to 24 hr. At each time point, 4 snapshots containing 30 microbeads each were imaged, and the bead sizes were measured. The scale bars represent 200 μm

The capability of partially crosslinked GelMA microgels to retain their shape in an aqueous medium at 37°C is a prerequisite for developing in situ forming annealed microgel scaffolds. To investigate the stability of partially crosslinked GelMA microbeads at the body temperature, they were transferred from the oil phase to DPBS and incubated at 37°C. Figure 2b shows the short‐time size evolution of semi‐photocrosslinked GelMA microbeads at 37°C as a function of photocrosslinking time. When uncrosslinked GelMA microgels were directly incubated at 37°C, they showed a strong sensitivity to temperature and started swelling significantly after 1 min of incubation, followed by complete dissolution in less than 7 min. The semi‐photocrosslinking process, however, led to time‐dependent stability of microgels, directly regulated by the photocrosslinking time. After 60 s of in‐oil photocrosslinking, GelMA microbeads showed a significant increase in their short‐term thermostability, with the majority of the particles (~ 70%) being able to retain their shape at 37°C after 5 min. Increasing the crosslinking time to 90 and 120 s increased the percentage of stable beads to >90 and >95%, respectively. Interestingly, the photocrosslinked GelMA microbeads did not undergo significant swelling or shrinking (Figure 2b). We also investigated the long‐term stability of the semi‐photocrosslinked GelMA microgels. Figure 3 shows the thermal stability of photocrosslinked microgels incubated at 37°C in DPBS for up to 24 hr. As can be seen in this figure, the GelMA microbeads photocrosslinked for 60 to 120 s were able to hold their shape and remain stable at body temperature for at least 24 hr without undergoing any significant swelling, shrinking, or dissolution.

**FIGURE 4:**
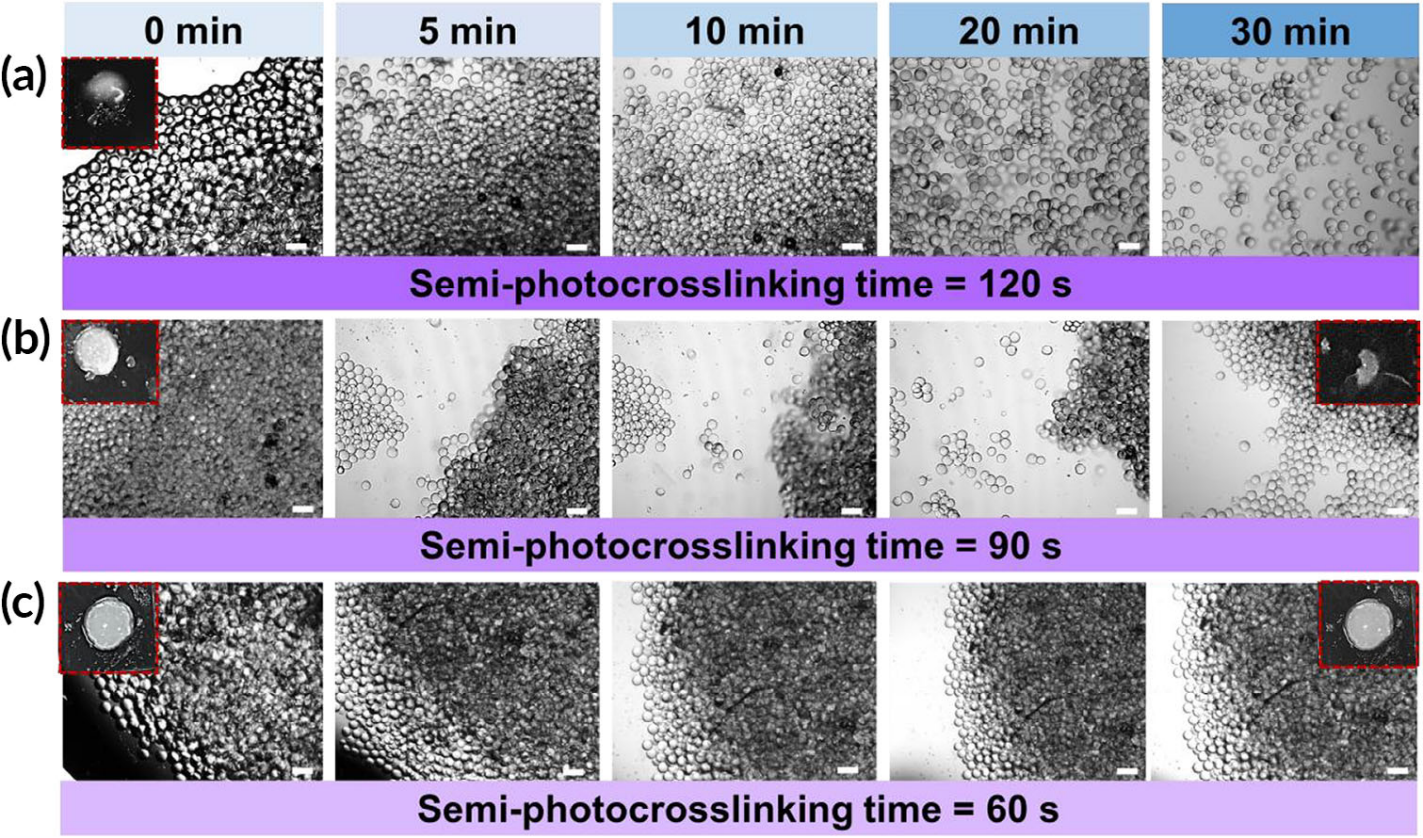
Assembly (annealing) of semi‐photocrosslinked gelatin methacryloyl (GelMA) microgels by ultraviolet (UV) light. Microgels semi‐photocrosslinked for (a) 120 s, (b) 90 s, or (c) 60 s were packed via centrifugation and annealed for 2 min using UV light with an intensity of ~10 mW/cm^2^, followed by assessing their stability after immersion in Dulbecco's phosphate‐buffered saline (DPBS) as a function of the time via optical imaging. The. Scale bars represent 200 μm

The capability of semi‐photocrosslinked GelMA microgels to undergo annealing and form a 3D scaffold was investigated as a function of photocrosslinking time in Figure 4. The GelMA microbeads were densely packed via centrifugation to ensure bead–bead contact, followed by annealing using UV light exposure at an intensity of ~10 mW/cm^2^ for 2 min. To assess scaffold stability, DPBS was added to the annealed hydrogels, followed by incubation at 37°C and optical imaging. Figure 4a presents the optical images of annealed scaffolds made up of GelMA microgels that were semi‐photocrosslinked for 120 s. These scaffolds were not stable and upon DPBS addition the annealed scaffolds broke apart. In fact, 120 s of photocrosslinking individual microgels inhibited the annealing process due to the consumption of crosslinkable methacryloyl groups. In addition, a higher crosslinking time leads to stiffer microbeads, and the stiffer the microbeads the more difficult the annealing process because the beads may not have sufficient contact surface area between them (they cannot deform as much to increase contact surface area). Reducing the microgel crosslinking time from 120 to 90 s permitted their partial annealing after the second UV light exposure. Figure 4b shows the scaffolds fabricated from microgels investigated the physical and biological properties initially photocrosslinked for 90 s, which underwent partial disintegration in less than 5 min postincubation in DPBS. Figure 4c shows the integrity of scaffolds fabricated from the annealing of GelMA microgels semi‐photocrosslinked for 60 s, yielding no significant microgel detachment from the scaffold within 30 min of incubation in DPBS at 37°C. Only when the semi‐photocrosslinking time of microgels was reduced to 60 s, they remained linked to each other post‐annealing, and a stable annealed particle scaffold was obtained.

**FIGURE 5:**
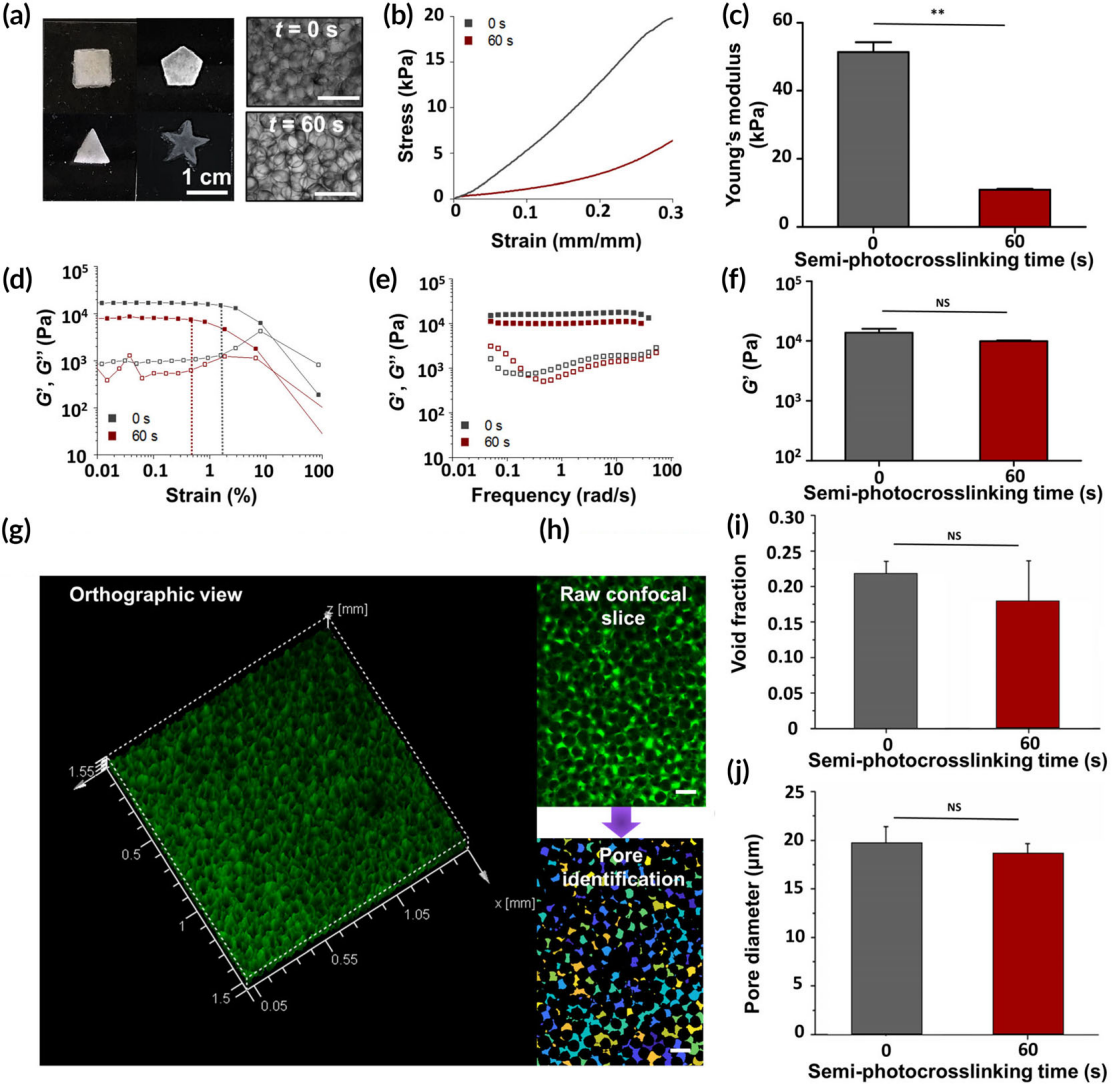
Space‐filling capaphysical and biological properties of stablebility, mechanical/rheological properties, and porosity of microporous hydrogels fabricated via annealing thermostable gelatin methacryloyl (GelMA) microgels. (a) Suspensions of semi‐photocrosslinked GelMA (20% wt/vol) microgels adapt to the shapes of polydimethylsiloxane (PDMS) molds and form multi‐layer microporous annealed‐particle scaffolds post‐ultraviolet (post‐UV) light exposure. (b) Compressive stress versus strain for the beaded scaffolds fabricated by annealing semi‐photocrosslinked (UV exposure time = 60 s, intensity ~100 mW/cm^2^) or physically crosslinked (UV exposure time = 0 s) GelMA microgels via 120 s of UV light exposure at an intensity of 10 mW/cm^2^ and their corresponding (c) compressive moduli. (d) Oscillatory strain sweep (at frequency = 1 rad/s) and (e) angular frequency sweep (at strain = 0.1%) tests to study the rheological properties of beaded scaffolds made up of semi‐photocrosslinked or physically crosslinked GelMA microgels. (f) Average storage modulus of beaded scaffolds at an angular frequency ~ 1 rad/s and strain ~0.1%. (g) Three‐dimensional (3D) confocal projection of a beaded scaffold made up of GelMA microgels semi‐photocrosslinked for 60 s. Void spaces was imaged by incubating the scaffold in high‐molecular weight fluorescein isothiocyanate (FITC)‐labeled dextran. (h) Scaffold pore diameter and void fraction were assessed by detecting the void spaces in two‐dimentional (2D) slices using a custom‐built MATLAB algorithm. Circles of equal area were fitted to the void area to estimate the equivalent diameter of pores. (i) Void space fraction and (j) median pore diameter for the GelMA microporous scaffolds made up of semi‐photocrosslinked or physically crosslinked GelMA microgels

We investigated the physical and biological properties of stable annealed particle scaffolds fabricated from the microgels semi‐photocrosslinked for 60 s. Shape adaptation of microgel suspensions was investigated by injecting them into polydimethylsiloxane (PDMS) molds with difference geometries (Figure 5a), showing that the semi‐photocrosslinked microgels are capable of holding their contact postinjection and undergoing photo‐annealing to form densely packed, mechanically stable microporous scaffolds, similar to nonphotocrosslinked microgels. Since implanted hydrogels are typically exposed to mechanical stress in vivo, we studied the mechanical properties of the microporous scaffolds and compared them with those of scaffolds prepared from physically crosslinked (nonphotocrosslinked) microgels. Figure 5b presents the stress–strain curves of the annealed scaffolds undergoing compression, which shows that at a given compressive strain, the compressive stress in scaffolds made up of semi‐photocrosslinked microgels is significantly lower than the scaffolds made up of physically crosslinked microgels. The compressive modulus, measured from a linear fit to the stress–strain curves at strain <10% is presented in Figure 5c, showing that the semi‐photocrosslinked beaded scaffolds have 4–5 times lower compressive modulus than the annealed scaffolds made up of physically crosslinked microgels.

The rheological properties of GelMA beaded scaffolds are presented in Figure 5d–f. The storage (*G′*) and loss (*G"*) moduli of beaded scaffolds were measured versus oscillatory shear strain (Figure 5d) at a constant frequency (1 rad/s) or versus angular frequency at a constant oscillatory shear strain (0.1%) (Figure 5e). The strain sweep experiments (Figure 5d) show that the strain limit of linearity based on the linear viscoelastic (LVE) region is about 0.5% for the scaffolds prepared from semi‐photocrosslinked beads and below 2% for the scaffolds prepared from physically crosslinked beads. A significant decrease in *G′* at strain >10% is observed in Figure 5d, suggesting similar brittle fracture for both types of scaffolds under oscillatory shear. Both beaded scaffolds prepared from semi‐photocrosslinked or physically crosslinked microgels behaved as elastic gels with *G′* independent of the applied frequency up to 10 rad/s and *G′* attaining values approximately one order of magnitude larger than *G″* (Figure 5e). In addition, the magnitude of each modulus was similar for both types of beaded constructs. As an example, Figure 5f presents the storage moduli of beaded scaffolds prepared using microgels that semi‐photocrosslinked for 0 s (physically crosslinked) or 60 s, which are not significantly different.

To enable the visualization and quantification of the void spaces among the annealed beads in each scaffold, a high‐molecular weight fluorescent dextran solution that is not able to penetrate the microgels was used to fill the interstitial space. Figure 5g presents the 3D projection of a semi‐photocrosslinked beaded GelMA scaffold from the orthographic view with void spaces in green. The diameter distribution of equivalent circles filling the void spaces was measured via analyzing the *z* stacks (Figure 5h). The void fraction of these scaffolds is compared with the ones fabricated from physically crosslinked microgels in Figure 5i, which shows that the chemical stabilization (semi‐photocrosslinking) of GelMA microbeads for 60 s before the annealing process had no significant effect on the void fraction attaining a value ∼20% for all scaffolds. In addition, both types of beaded scaffolds had median pore diameter of ~20 μm, as shown in Figure 5j. This indicates that for both approaches there are interconnected void spaces that may enable migration and proliferation of cells.

**FIGURE 6:**
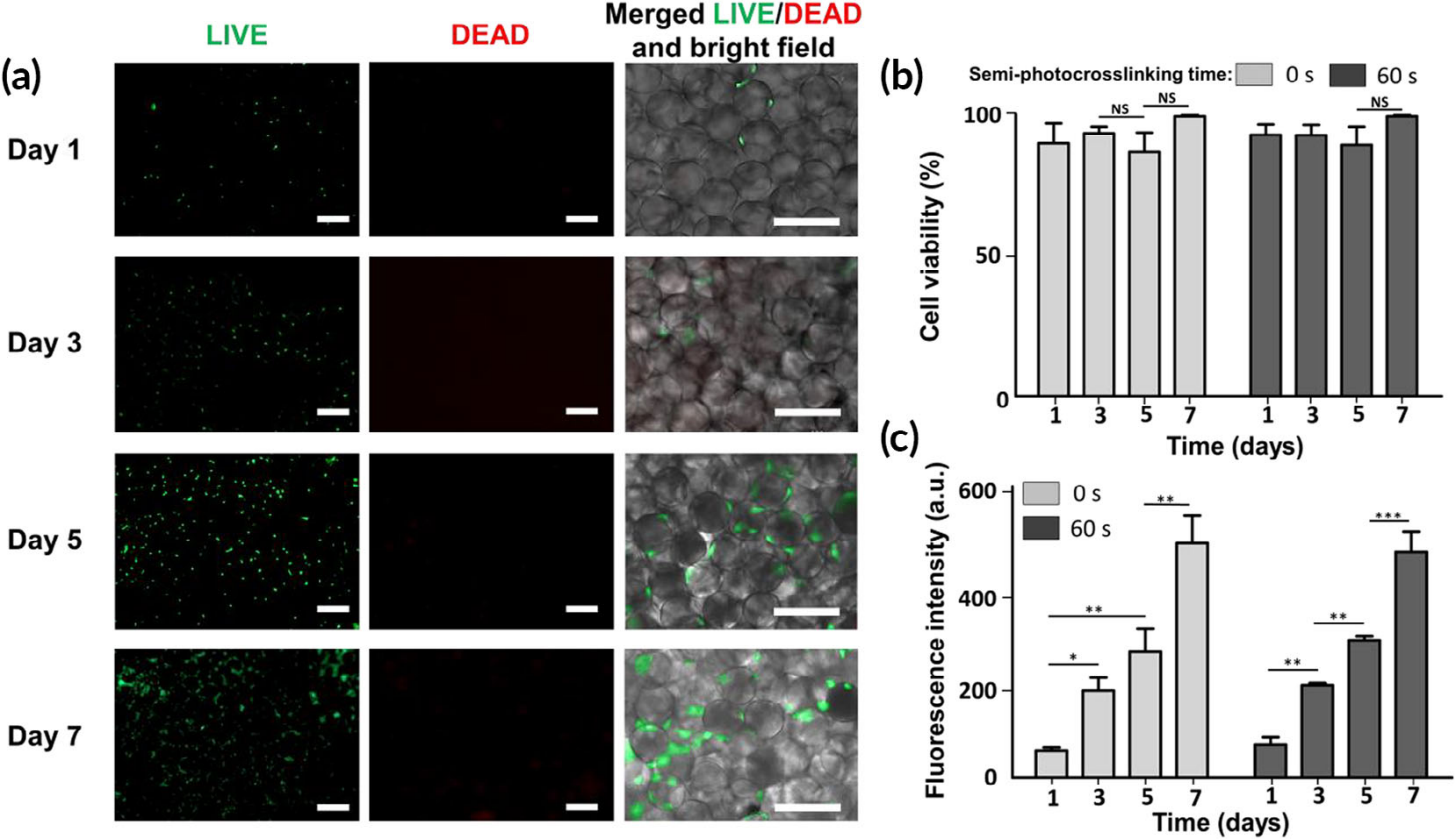
In vitro biological activities of microporous hydrogels fabricated via annealing thermostable gelatin methacryloyl (GelMA) microgels. (a) Assessment of live (green) and dead (red) NIH/3T3 cells after 1, 3, 5, and 7 days of culture in three‐dimentional (3D) beaded GelMA scaffolds prepared from photo‐annealing semi‐photocrosslinked microgels (ultrabviolet [UV] exposure time = 60 s, intensity ~100 mW/cm^2^) via 120 s of UV light exposure at an intensity of ~10 mW/cm^2^. Brightfield images show the spreading of cells among GelMA microbeads. (b) Cell viability was measured based on the number of live cells divided by the total cell number in beaded GelMA scaffolds fabricated from semi‐photocrosslinked (UV exposure time = 60 s) or physically crosslinked (UV exposure time = 0 s) GelMA microgels photo‐annealed via 120 s of UV light exposure at an intensity of ~10 mW/cm^2^.(c) Metabolic activity of the cells embedded in the 3D beaded scaffolds as a function of incubation time, measured using the PrestoBlue® assay. Scale bars represent 200 μm

The in vitro biological activity of microporous GelMA scaffolds was studied by mixing NIH/3T3 fibroblast cells with the semi‐photocrosslinked GelMA beads, followed by the UV light exposure for 120 s at 10 mW/cm^2^ to form cell‐laden microporous constructs. As a control, the biological activity of cell‐laden GelMA microporous scaffolds fabricated by the photo‐annealing of physically crosslinked microgels was also investigated. Figure 6a shows the live/dead cell viability assay of annealed cell‐laden scaffolds fabricated from semi‐photocrosslinked microgels mixed with cells within 7 days of culture. As can be seen in this figure, cells readily fill the interconnected microscale pores and adhere to the beads, followed by spreading and proliferation in 7 days. Cell viability, shown in Figure 6b, was quantified by normalizing the number of live cells with the total cell number. Both beaded scaffolds, regardless of the semi‐photocrosslinking condition, afforded cell viability ~100%. At such a high polymer concentration (20% wt/vol), the bulk (nanoporous) GelMA scaffolds are not able to support cell viability.^1^ The metabolic activity of the cells encapsulated among the GelMA microbeads was measured using the PrestoBlue® assay (Figure 6c), which showed a ~2.8‐, 4.2‐, and 7‐fold increase 3, 5, and 7 days post seeding, respectively. Importantly, no significant difference was observed between the microporous hydrogels fabricated by the photo‐annealing of semi‐photocrosslinked or physically crosslinked GelMA microbeads.

## 3 DISCUSSION

In our previous works,^8^, ^20^ we introduced the fabrication of beaded GelMA scaffolds from the photo‐annealing of physically stabilized GelMA microgels in an aqueous phase. The physical stabilization of GelMA microgel was conducted via the temperature‐mediated phase transition (triple helix formation) of gelatin (denatured collagen I) at a low temperature (e.g., 4°C). In this approach, GelMA microbeads must be maintained at 4°C during purification (oil/surfactant removal and transfer to an aqueous phase) and in vitro or in vivo annealing, which typically demands a long period increasing the probability of bead melting. Such a long processing time may impose several limitations to the biomedical applications of GelMA microgels, mainly during cell culture and in situ scaffold formation. Here, we engineered thermostable GelMA microbeads via UV‐light‐mediated semi‐photocrosslinking and investigated their stability and annealing capability, as well as the properties of annealed microgel scaffolds. Importantly, we identified a critical range of crosslinking parameters that facilitated successful annealing of microgel scaffolds. The semi‐photocrosslinking of microbeads by UV exposure (60 s or more, intensity ~100 mW/cm^2^), performed in the oil phase, chemically stabilized the microgels, preventing them from melting during the oil/surfactant removal process and bead transfer to an aqueous medium. Thus, these purification steps were readily performed at room temperature.

The photocrosslinking of beads performed in a fluorocarbon oil is susceptible to oxygen inhibition due to the quenching of otherwise initiating/propagating radicals via forming peroxyl radicals. Peroxyl radicals do not initiate the conversion of vinyl groups, and the radical polymerization of methacryloyl groups is impaired until the dissolved oxygen is fully consumed. The competition between the diffusion and the consumption of oxygen is clearly observed during in‐oil microbead crosslinking: after the UV curing in the oil phase, the droplets exhibit a polymerized core and an unpolymerized or poorly polymerized shell^28^, ^33^, ^34^ that is partially dissolved when the beads are transferred to an aqueous medium, decreasing the bead diameter by ~10% (~10 μm) (Figure 2a). Semi‐photocrosslinking time regulates the methacrylate conversion. Accordingly, we selected a range for UV curing time (60–120 s) to obtain partially crosslinked microbeads, followed by bead purification and incubation at 37°C in an aqueous phase. Photocrosslinking of GelMA for 60–120 s results in a degree of crosslinking ranging from ~80% to more than 95%.^35^ Stable, partially photocrosslinked GelMA microbeads retain their shape at 37°C with no significant change in diameter (Figures 2b and 3).

To assess the annealing capability of partially photocrosslinked microgels, they were packed via centrifugation, pipetted into a mold using a positive displacement pipette to maintain their contact proximity, and photo‐annealed to generate 3D scaffolds. There exists a tradeoff between the semi‐photocrosslinking time (regulating microgel thermostability) and microgel annealing capability. When GelMA microbeads are not semi‐photocrosslinked, they are thermally unstable (melt at 37°C); however, they form the strongest and most stable annealed particle scaffolds because of the availability of all methacryloyl groups and potentially due to a higher contact area between the softer, physically crosslinked beads. As the semi‐photocrosslinking time increases, the thermostability of beads is improved; however, their capability to chemically link to each other (anneal) decreases due to the partial consumption (prepolymerization) of methacryloyl groups, which would otherwise chemically bind the beads together during photo‐annealing. A semi‐photocrosslinking time of 120 s completely impairs bead annealing because almost all methacryloyl moieties react before annealing. The reduction of the semi‐photocrosslinking time to 90 s and 60 s yields more free methacryloyl moieties, enabling the formation of 3D scaffolds from microgel annealing through a secondary UV‐light exposure (Figure 4). Although both exposure periods (60 s and 90 s) lead to the formation of 3D scaffolds, only the semi‐photocrosslinking time of 60 s results in stable scaffolds that do not disintegrate in DPBS.

The semi‐photocrosslinking of GelMA microgels has a significant effect on the compressive modulus of 3D‐annealed particle scaffolds. Under a normal (perpendicular) load, the scaffolds made up of semi‐photocrosslinked (60 s) GelMA microgels are much softer (4‐5 times lower compressive modulus) than the scaffolds fabricated from physically crosslinked beads; however, the failure strain for both of them is similar. This is an interesting fact about microporous annealed GelMA (and other similarly fabricated) scaffolds that while the building blocks may have similar mechanical properties as a result of similar polymer concentration and crosslinking condition, the annealed scaffold may attain a broad range of mechanical properties as a result of tailored bead–bead interactions and binding strength. Furthermore, under oscillatory shear, both scaffolds behave similarly: the trend of viscoelastic moduli versus strain or frequency sweeps is identical, and the storage and loss moduli of scaffolds at a low strain or frequency are not statistically different. Importantly, the void fraction and median pore diameter in both scaffolds are also similar, showing that the semi‐photocrosslinking of beads does not affect the pore microstructure in the annealed particle scaffolds because thermostable microgels do not undergo melting and filling the void spaces (Figure 5).

In vitro biological assessments of 3D annealed particle scaffolds prepared from semi‐photocrosslinked GelMA microgels confirm their capability in supporting a high cell viability, spreading, and proliferation within at least 7 days (Figure 6). The results confirm that there is no significant in vitro cellular activity difference between the microporous scaffolds fabricated from semi‐photocrosslinked or physically crosslinked microgels. Such similar results are favorable and may be explained by considering the similar void fraction and median pore diameter values of both beaded scaffolds. In addition, the semi‐photocrosslinked scaffold can hold their structural integrity during the cell culture procedure, including cell seeding, photo‐annealing, incubation at 37°C, imaging, and viability/metabolic activity assessments. Our novel method to generate thermostable, annealable GelMA microbeads for the fabrication of in situ forming microporous hydrogel scaffolds may provide new opportunities for advanced tissue engineering applications in which temperature must be maintained as close to the body temperature as possible.

## 4 CONCLUSIONS

We introduced a facile route for the fabrication of in situ forming microporous hydrogel scaffolds using thermostable annealable GelMA microgel building blocks. GelMA microbeads, generated as a water‐in‐oil emulsion, were semi‐photocrosslinked in an oil phase, imparting thermostability to the building blocks, which were purified at room temperature and annealed at physiological temperature. We showed that increasing the semi‐photocrosslinking time of GelMA microgels increased thermostability while compromising the annealing capability of building blocks. Accordingly, the semi‐photocrosslinking condition was optimized to yield thermostable GelMA microgels that were well annealable, forming microporous scaffolds at physiological temperature. This technology expands the biomedical applications of gelatin‐based microporous annealed‐particle scaffolds, which may be extended to other thermosensitive biomaterials, setting the stage for developing thermostable ECM‐mimetic in situ forming microporous hydrogel scaffolds.

## 5 EXPERIMENTAL

### 5.1 Materials

Silicon wafers were purchased from University Wafer (Boston, MA), negative photoresist KMPR 1050 was purchased from MicroChem Corp. (Boston, MA), and the microfluidic chips were fabricated using PDMS base and its curing agent (SYLGARD™ 184 Elastomer Kit, Dow Corning, MI). The microfluidic tubing were 1569‐PEEK Tubing Orange 1/32“ OD × .020” ID (IDEX Corp., Lake Forest, IL) and Tygon Flexible Plastic Tubing 0.02“ ID × 0.06” OD (Saint‐Gobain PPL Corp., Garden Grove, CA). The microfluidic device was treated with Aquapel® Glass Treatment (Pittsburgh Glass Works LLC, Pittsburgh, PA). 3M™ Novec™ 7500 Engineered Fluid (Novec 7500 oil) was purchased from 3M (St. Paul, MN). Photoinitiator 2‐hydroxy‐1‐(4‐[2‐hydroxyethoxy]phenyl)‐2‐methylpropan‐1‐one (Irgacure 2959), gelatin from porcine skin (Type A, 300 bloom), methacrylic anhydride (MA, 94%), 1H,1H,2H,2H‐perfluoro‐1‐octanol (PFO, 97%), and fluorescein isothiocyanate (FITC)–dextran (500 kDa) were procured from Sigma‐Aldrich (St Louis, MO). Milli‐Q water (electrical resistivity ~18.2 MΩ cm at 25°C) was from Millipore Corporation. Dialysis membranes (molecular weight cutoff ~12–14 kDa) were purchased from Spectrum Lab Inc. (Milpitas, CA). Cover slips (No. 1) and VistaVision™ Microscope Slides (Plain 3″ × 1″) were provided by VWR (Monroeville, PA), and microscope glass slides (18 mm × 18 mm × 300 μm) were purchased from Thermo Fisher Scientific (Millersburg, PA). Pico‐Surf™ 1 (5% [wt/wt] in Novec™ 7500) was provided by Sphere Fluidics Inc (Cambridge, UK). DPBS solution (1×), Dulbecco's modified Eagle's medium (DMEM) + GlutaMAX (supplemented with high glucose and pyruvate), penicillin–streptomycin (P/S, 100×), heat‐inactivated fetal bovine serum (HI FBS) and trypan blue were from Gibco (New York, NY). Live/dead™ viability/cytotoxicity kit and PrestoBlue® were purchased from Thermo Fisher Scientific.

### 5.2 Methods

#### 5.2.1 Microfluidic device fabrication

To generate uniform‐sized spherical microbeads, a high throughput step emulsification microfluidic device for producing water‐in‐oil emulsion, was fabricated as previously reported.^13^ Briefly, master molds were fabricated using a two‐layer photolithography process. The first and the second layers determine the nozzle channel height (32 μm) and the height of inlet/outlet channels in the reservoir region (160 μm), respectively. The first layer was added by spin coating KMPR 1025 on mechanical grade silicon wafers (4 in) and soft baking following manufacture protocol. A photo‐transparency mask (CAD/Art Services) was then used to pattern the nozzle layer using a mask aligner (Karl Suss MA6) to expose with UV light (30 s @ 12 mW/cm^2^). The nozzles were 20 μm wide and taper to 150 μm at the outlet channel (Figure 1). Following the patterning step, wafers were treated by a postexposure bake. A second layer was then added on top of the first layer by spin coating KMPR 1050 and soft baking. A second mask was used to pattern the inlet and outlet channels following the same process. Unpatterned photoresist was removed using SU8 developer to reveal the final master molds. To fabricate the PDMS devices, PDMS base and the curing agent were mixed at a 10 to 1 ratio and poured onto the molds affixed to petri dishes, followed by vacuum degassing and curing in an oven (65°C for >4 hr). The PDMS devices were detached from the mold, and holes were punched (diameter ~ 0.8 mm) at the inlets and outlets using a biopsy punch. To seal the microchannels, the device and a glass slide were activated via air plasma for 40 s (Plasma Cleaner, Harrick Plasma, Ithaca, NY) and bonded together. To render the channel surfaces fluorophilic, the device was treated with Aquapel®, followed by rinsing with the Novec 7500™ oil. Finally, the devices were placed in an oven at 70°C for 1 hr to evaporate the residual oil in the channels.

#### 5.2.2 Synthesis of GelMA

We synthesized GelMA based on our protocol previously published.^22^, ^23^ Briefly, 10 g of porcine skin gelatin (Type A) was dissolved in 100 mL of DPBS at 50°C, followed by adding 8.0 mL of MA dropwise. The mixture was reacted for 2 hr under magnetic stirring at 50°C after which the reaction was stopped with a twofold dilution of warm DPBS, followed by dialysis for at least 1 week using a membrane (12–14 kDa Mw cutoff) at 40°C to remove impurities, such as unreacted MA. Finally, the solution was freeze‐dried to yield a white foam and stored at room temperature before using for microgels fabrication.

#### 5.2.3 GelMA bead fabrication

Freeze‐dried GelMA was dissolved in a mixture of DPBS and a photoinitiator (0.5% wt/vol, Irgacure 2959) at 80°C for ~10 min to yield GelMA solutions (20% wt/vol). To prepare GelMA beads, the GelMA solution was used as a dispersed phase, while 0.5 wt% PicoSurf in Novec 7500 oil was used as a continuous phase to prepare a water‐in‐oil emulsion. Both phases were individually injected into the inlet microchannels of a step emulsification microfluidic device using syringe pumps (Harvard Apparatus PHD 2000, Holliston, MA). The flow rate of the continuous phase was maintained constant at 100 μL/min, while the flow rate of the dispersed phase was adjusted to obtain droplets of ~100 μm. The surfactant‐stabilized GelMA droplets were collected in a microcentrifuge tube and stored at 4°C to induce physical gel formation in oil. The GelMA microsphere were imaged using an inverted microscope equipped with a camera (Axiocam 503 mono, 60 N‐C 1″ 1,0X), their microstructure was visualized using brightfield microscopy (Axio Observer 5, Zeiss, Germany), and their size was determined using image J software (Version 1.52e, National Institute of Health).

#### 5.2.4 Fabrication of beaded GelMA scaffolds from semi‐photocrosslinked GelMA microbeads

To semi‐photocrosslink GelMA beads, they were first produced as an aqueous phase‐in‐oil emulsion, followed by UV light exposure (360–480 nm, Omnicure, Excelitas, Pleasanton, CA) for different times (60, 90, and 120 s) at an intensity of the staining solution containing ~100 mW/cm^2^. Beads were then separated from the oil using a 20% PFO solution in Novec™ 7500 oil (1:1 volume ratio) to break the emulsion, and then transferred to an aqueous phase (DPBS). The oil‐free microbeads were rinsed with a DPBS‐photoinitiator mixture (0.5% wt/vol, Irgacure 2959), followed by pulse centrifugation at 6000 rpm for 10 s to pack the beads at the bottom of the container. The washing step was repeated twice before placing the bead suspension in the incubator at 37°C for 30 min. Then, the semi‐crosslinked GelMA microbeads were washed one more time with DPBS containing 0.5% wt/vol of photoinitiator and centrifuged at 6000 rpm for 20 s for packing. Finally, using a positive displacement pipette, the concentrated microbead suspension was transferred into a PDMS mold (diameter ~8 mm, height ~1 mm) and UV cured for 2 min at ~10 mW/cm^2^, yielding fully chemically crosslinked and annealed microgels.

#### 5.2.5 Fabrication of beaded GelMA scaffolds from physically crosslinked GelMA microbeads

To fabricate beaded GelMA scaffolds from physically crosslinked GelMA microbeads, a previously reported protocol was used.^8^, ^20^ Briefly, after microbead generation using the microfluidic device (described in Section 5.2.3), the excess oil was separated from the microbead emulsion using a pipette. A solution of 20% PFO in Novec™ 7500 oil was added to the bead suspension (1:1 volume ratio) to destabilize the emulsion and transfer the beads to the aqueous phase at 4°C. The oil‐free microbeads were washed using cold DPBS (4°C) containing the photoinitiator (0.5% wt/vol, Irgacure 2959), followed by pulse centrifugation at 6000 rpm for 10 s to pack the beads. The washing step was repeated twice. Finally, using a positive displacement pipette, the concentrated microbead suspension was transferred into a PDMS mold (diameter ~8 mm, height ~1 mm), followed by two UV light (360–480 nm, Omnicure, Excelitas) exposure steps (first exposure intensity ~100 mW/cm^2^ for 60 s; second exposure intensity ~10 mW/cm^2^ for 2 min), yielding chemically photocrosslinked and annealed microgels. The annealing condition was selected in a way that mimics the fabrication of beaded GelMA scaffolds from the semi‐photocrosslinked GelMA microbeads.

#### 5.2.6 Pore size and void space fraction measurement

Scaffolds were incubated in a FITC–dextran (Mw = 500 kDa) solution (15 mM) to visualize the void spaces. The dye‐infused scaffolds were imaged using a Leica inverted SP5 confocal microscope (Germany). For each sample, 77 *z*‐slices were captured to cover a total height of ~150 μm. Median pore diameter and void space fraction were measured using a custom developed Matlab code (Matlab, version 2017b). The stacked images were converted into discrete regions using an adaptive thresholding, and the void space fraction was calculated based on the voxel volume of void space regions. In addition, average pore diameter was measured based on a previously published method.^12^

#### 5.2.7 Mechanical analyses

For compression tests, scaffolds were crosslinked in disk PDMS molds (diameter ∼8 mm and height ∼1 mm) and incubated in DPBS for 1 hr at room temperature. We conducted compression tests on the hydrated (wet) samples using an Instron mechanical tester (Instron 5542, Norwood, MA) at the rate ∼1 mm/min. The linear stress–strain region was fitted with the best line at strain <10%, and the slope was registered as the compression modulus (= stress/strain).

#### 5.2.8 Rheological analyses

Oscillatory shear rheological assessments were conducted using an MCR 302 Rheometer (Anton Paar, Graz, Austria). We used a parallel plate geometry (diameter = 8 mm, sandblasted measuring plate, PP08/S) to load the samples, followed by setting the equilibration temperature to room temperature. To measure the viscoelastic moduli, oscillatory frequency sweep tests were conducted at 0.1–100 rad/s under a small oscillatory strain ∼0.1% (LVE region) at 25°C. The hydrogel scaffolds were hydrated during the experiments (total time ~20 min) and maintained in an enclosed chamber. The viscoelastic moduli versus oscillatory strain (0.01–100%) were also registered at a frequency ~1 rad/s.

#### 5.2.9 In vitro biological activities of cell‐laden scaffolds

This section includes NIH/3T3 fibroblast cell culture, 3D cell encapsulation in microporous hydrogel scaffolds, metabolic activity assessment, and live/dead assay.

##### Cell culture

Fibroblast (NIH/3T3) cells were cultured in cell culture flasks placed in a standard cell culture incubator (5% CO_2_ atmosphere at 37°C, Thermo Fisher Scientific). The cells were cultured in sterile DMEM + GlutaMAX media, supplemented with 10% FBS and 1% P/S and passaged twice a week. The cell culture medium was replaced every 2 days. To assess the in vitro cellular function of fibroblasts, they were trypsinized (0.5% trypsin–ethylenediaminetetraacetic acid (EDTA)), followed by counting using a hemocytometer, resuspension in a small volume (15 μL), and mixing with GelMA beads to prepare beaded‐scaffolds from semi‐photocrosslinked beads or physically crosslinked GelMA microgels. The semi‐crosslinked or physically crosslinked microgels were prepared under sterile conditions (using sterile solutions, in a biosafety cabinet) and were mixed with cells at 37°C or 4°C, respectively, followed by UV light‐mediated annealing (exposure time = 120 s, intensity = 10 mW/cm^2^). The resulting cell‐laden scaffolds were maintained under a 5% CO_2_ atmosphere at 37°C for 2 hr to facilitate cell adhesion, followed by gentle addition of 1 mL of fresh medium and culturing. The medium was replaced every 2 days.

##### Cell viability

We evaluated the survival rate of cells embedded in the GelMA microporous 3D scaffolds after 1, 3, 5, and 7 days of culture. Cell viability was assessed using a standard live/dead™ assay (calcein AM/ethidium homodimer) according to the manufacturer's instructions. To perform the assay, 500 μL of the staining solution containing 0.25 μL of calcein AM and 1 μL of ethidium homodimer in DPBS was used to replace the cell culture medium, followed by incubation in the dark at 37°C for 20 min. Live and dead cells were imaged using an Axio Observer 5 fluorescent microscope (Zeiss, Germany) equipped with Axiocam 503 mono (60 N‐C 1″ 1,0X) camera at excitation/emission wavelengths ∼494/515 nm for calcein (green, live cells) and 528/617 nm for ethidium homodimer‐1 (red, dead cells). NIH ImageJ software was used to quantify the the cell viability based on the ratio of the live cell number to the total number of cells.

##### Metabolic activity assessments

The metabolic activity of cells was measured after 1, 3, 5, and 7 days of culture using the PrestoBlue® cell viability reagent according to the manufacturer's protocol. PrestoBlue® solution was made by adding 1 mL of PrestoBlue® solution (10×) to 9 mL of the cell culture media. The initial cell culture media were replaced with 500 μL of the PrestoBlue® solution, and the cell‐laden scaffolds were incubated in the dark at 37°C for 1 hr. The fluorescence intensity was measured using a microplate reader (excitation ∼530 nm and emission ∼590 nm, BioTek UV/vis Synergy 2, Winooski, VT), which was corrected based on the background signal originated from the cell‐free media containing a similar PrestoBlue® dye concentration.

#### 5.2.10 Statistical analysis

We conducted the measurements at least in triplicate and reported the data as mean values ± *SD*. In addition, we performed statistical analyses based on the one‐way analysis of variance, followed by the Bonferroni comparison test to identify the statistically significant differences based on the *p* values: *p < .05, **p < .01, ***p < .001, ****p < .0001.

## CONFLICT OF INTERESTS

The authors declare no potential conflict of interest.
